# Population based retrospective cohort study on risk of retinopathy of prematurity in twins

**DOI:** 10.1371/journal.pone.0230346

**Published:** 2020-03-12

**Authors:** Hui-Chen Tseng, Fung-Chang Sung, Chih-Hsin Mou, Hsiang Yu Lin, Chun-Chi Chiang, Ning-Yi Hsia, Ya-Ling Tzeng

**Affiliations:** 1 School of Nursing and Graduate Institute of Nursing, China Medical University, Taichung, Taiwan; 2 Department of Nursing, China Medical University Hospital, Taichung, Taiwan; 3 Management Office for Health Data, China Medical University Hospital, Taichung, Taiwan; 4 Department of Health Services Administration, China Medical University College of Public Health, Taichung, Taiwan; 5 Department of Food Nutrition and Health Biotechnology, Asia University, Taichung, Taiwan; 6 Department of Neonatolgy, China Medical University Children’s Hospital, Taichung, Taiwan; 7 Department of Ophthalmology, China Medical University Children’s Hospital, Taichung, Taiwan; Medical University of Vienna, AUSTRIA

## Abstract

**Background:**

Twin infants are likely at great risk for ROP, but studies reported conflicting findings and population studies examining the risk of retinopathy of prematurity (ROP) in twins is limited. We aimed to evaluate the ROP risk in the cohort of one of twins, comparing to singletons.

**Material and methods:**

Using insurance claims data of a half of children in Taiwan ages 18 and less, we established a twin cohort (N = 27830) born in 1998–2009 and a randomly selected singleton cohort (N = 111080) frequency matched by sex, birth year, residential area and parental occupation and followed up to 2012 years.

**Results:**

The overall incidence rate of ROP was 13.6-fold greater in the twin cohort than in the singleton cohort (35.1 vs. 2.58 per 10,000 person-years; adjusted HR = 13.4, 95% CI = 11.7–15.3; p <0.0001). The ROP incidence was slightly higher in boys than in girls, higher in children in more urbanized areas and born to mothers without works. The incident ROP increased with decreasing birthweight. For children with birthweight <1000 grams, the ROP incidence was 1.2-fold greater in the twin cohort than in the singleton cohort (1243.2 vs. 1016.3 per 10,000 person-years). The use of mechanical ventilation was associated with increased ROP risk for both cohorts, particularly for infants who were under invasive treatment.

**Conclusion:**

Infants who were born as twins or born with low birthweight were at an elevated risk of developing ROP. Extreme cautious and close monitor are required for new born with low birthweight and have undergone with mechanical ventilation.

## Introduction

Retinopathy of prematurity (ROP) is an eye disorder characterized by neurovascular disruption in the immature retina and is known to lead to visual impairments and blindness. [1–4] This disorder affects mainly the preterm or low birthweight infants who have received oxygen supplementation during the early postnatal care[4–6]. Worldwide approximately 10–11% of infants are born with premature and 15% are born with low birthweight, vary among nations. [7–9] The risk of ROP varies mainly by birthweight and the availability of neonatal intensive care. Up to 50% of infants with birthweight <1500 g may develop ROP. [10] Prevalence rates of both premature and low birthweight are much greater in developing countries than in developed countries. In well developed countries, ROP is limited to extreme low birthweight infants. [11]

Furthermore, ROP is characterized by progressive degenerating retinal vasculature and is classified on the basis of location (ZoneⅠto Zone Ⅲ) and severity (stage 1 to stage 5) and extent.[12] In general, the more posterior the location, the greater extent of involvement; and the higher the stage, the more severe form of ROP is developed. [13] The incidence and severity of ROP vary among countries. The risk for infants with stage 5 ROP is greater in the population of less developed areas than developed areas. [14, 15] It is well known that low birthweight is a common consequence of premature birth, and premature birth is a common complication in multiple births. These infants are thus likely at a higher risk for ROP. However, studies have reported conflicting findings regarding the ROP risk in twin or multiple births. [16– 19] Stduies with small sample size have the weakness of low statistical power. An early Israel study using comparative case series for comparing ROP risk between infants of assisted conception versus natural conception, and 159 singleton versus multiple births (134 twins and 70 triplets and quadruplets), with birth weights of 1500 g or less. Surprisingly, singletons had a significant risk developing advanced ROP of stage II and Ⅲ than twins.[16] Two respropective studies, both reported no significant findings between multiparity and ROP risk. [17, 18] A Turkey study focussed on comparing ROP between 225 multiple births and 76 single births in extremely preterm infants with ≤27 weeks of gestational age at birth. [18] Another Hong Kong study compared 120 preterm infants in twins based on the order of delivery and found Twin 1s were more susceptible to the postnatal risks for ROP than the Twin 2s. [19] However, this study compared twin 1 and twin 2, rather than singletons versus twins. These disagreement in findings with small sample sizes and different study design may not be generalizable to the population of twins versus singletons in developing ROP. A recent study in Taiwan using insurance claims data found that the incident ROP was steady throughout the period from 2002 to 2011, whereas the treatment–requiring ROP was in an increasing trend. Results showed that, additionally low birth weight, multiparity was associated with developing ROP with an adjusted odds ratio 1.17 (95% confidence interval, 1.062–1.290). [20]

Because of disagreements in study designs, findings from these studies may not be generalizable to the population of twins. Population-based studies are encouraged to seek further detailed evaluation on the progress of ROP for infants of twins or multiple births comparing with singletons. We therefore used insurance claims data of Taiwan to compare the ROP risk between infants of twin births and singletons.

## Material and methods

### Study population

This study used a subset of claims data of the Taiwan National Health Insurance (TNHI) program, consisting of medical records of a half of all children ages 18 years and less randomly selected from all children in Taiwan. TNHI is a universal health insurance system, which has covered over 99 percent of population in Taiwan. [21] We obtained the database from the National Health Research Institutes, authorized by the Ministry of Health and Welfare. The data set consisted of demographic status of insured population and claims records of outpatient and inpatient cares, with information on treatment, medication and cost of care. Diseases were coded with the International Classification of Diseases, Ninth Revision, Clinical Modification (ICD-9-CM). All identifications of insured people had been scrambled and replaced with surrogate personal identifiers before the data set was released for purposes of privacy protection and data linkage.

**Fig 1:**
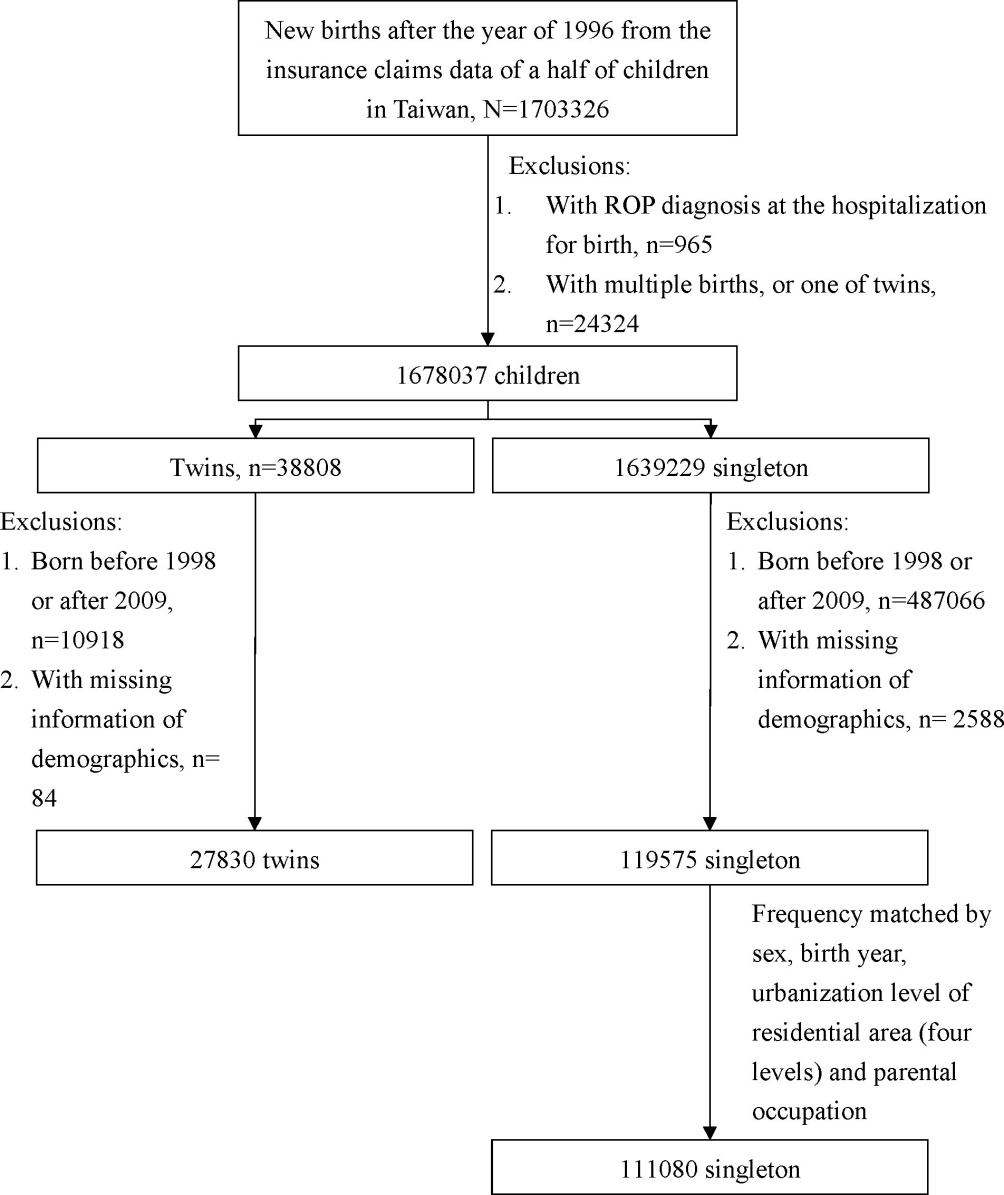
Flow chart of patients of two cohorts.

From the claims data of children (N = 1,703,326), we randomly selected one infant from each pair of twin birth (N = 38808), after excluding triplets and multiple births. Among them, infants born from 1998 to 2009 with demographic information were included as the twin cohort (N = 27830) (Fig 1). From the same database of children, we randomly selected a cohort of singleton infants with a size 4-fold of the twin cohort. The two cohorts were frequency matched by sex, birth year, urbanization level of residential area (four levels) and parental occupation (white collar, blue collar and other). Urbanization levels of residential areas were classified into seven levels, level “1” was the highest urban and level “7” was the lowest urban. We combined level “5–7” into level “4” because of smaller population sizes. Occupation of “white collar” referred to workers with longer indoor hours, such as government employees, office staffs, teachers, and so on. Occupation of “blue collar” referred to workers with longer outdoor hours, such as farmers, fishers, crews and so on. Occupation of “others” included house keepers, impoverished people, religious people, veterans, and so on. Infants of full-term birth and preterm birth were included.

### Data analysis

Frequency distributions of gender and birth year of infants, and their residential areas and parental occupations were expressed as categorical variables and examined using the Chi-square test between the two cohorts. The person-years of follow-up time was estimated for each infants from the birth date to the diagnosis of ROP, death, withdrawal from the insurance, or the end of 2012. The incidence density rates of ROP per 10,000 person-years were computed for each cohort by the demographic variables. The Cox proportional hazards regression analysis was used to calculate hazard ratios (HRs) and 95% confidence interval (CIs) of ROP for the twin cohort compared to the singleton cohort. We further calculated the incidence density rate of ROP by birthweight for both cohorts and calculated HRs compared to normal birthweight singletons. The incidence rates and HRs of ROP associating with the use of non-invasive and invasive mechanical ventilation with oxygen in the early postnatal care were also estimated. We categorized infant weight into 6 groups: < 1000 gram, 1000–1249 gram, 1250–1499 gram, 1500–1749 gram, 1750–1999 gram, and 2000–2499 gram for analysis purpose. We used the SAS statistical package (version 9.4; SAS Institute Inc., Cary, NC, USA) to conduct data analyses. The Research Ethics Committee at China Medical University and Hospital in Taiwan has approved the use of insurance claims data for the study (CMUH-104-REC2-115).

## Results

The study population included 111080 singletons and 27830 twin neonates. Table 1 shows that the baseline demographic distributions were similar in both cohorts, with more boy (51.6%), more births in 1998–2001, more urban residents and more parents with white collar jobs.

**Table 1:** Demographic status of singleton and twin cohorts.

	Singleton (N = 111080)	Twin (N = 27830)	
Variable	No.(%)	No.(%)	P-value
Sex			0.99
Girl	53804(48.4)	13481(48.4)	
Boy	57276(51.6)	14349(51.6)	
Birth year			0.98
1998–2001	50080(45.1)	12544(45.1)	
2002–2005	35796(32.2)	8973(32.2)	
2006–2009	25204(22.7)	6313(22.7)	
Urbanization			0.95
1	34824(31.4)	872(31.3)	
2	33996(30.6)	8514(30.6)	
3	21688(19.5)	5437(19.5)	
4	20572(18.5)	5158(18.5)	
Parental occupations			0.95
White collar	64300(57.9)	16093(57.8)	
Blue collar	17256(15.5)	4332(15.6)	
Others	29524(26.6)	7405(26.6)	

Chi-square test

The overall incidence rate of ROP was 13.5-fold greater in twins than in singletons (35.1 vs.2.6 per 10000 person-years), with an adjusted HR of 13.4 (95% CI = 11.7–15.3, P<0.0001) after controlling for sex, urbanization, and parental occupation (Table 2). The ROP incidence was slightly higher in boys than in girls, higher in children in more urbanized areas and born to parental occupation of “others”. In stratified analysis of sex, urbanization, and parental occupation, the twin cohort had about 10- to 15-fold higher ROP risk than the singleton cohort.

**Table 2:** Incidence of retinopathy of prematurity and Cox proportional hazards regression analysis estimated twin to singleton adjusted hazard ratio by demographic status.

	Twin			Singleton				
Demographic factors	Event1	Person-years	Rate†	Event	Person-years	Rate†	Adjusted HR(95% CI)	P-value
Overall	932	265377	35.1	282	1093904	2.6	13.4 (11.7–15.3)	<0.0001
Sex								
Girl	440	128631	34.2	134	530155	2.5	13.3 (11.0–16.2)	<0.0001
Boy	492	136746	36.0	148	563749	2.6	13.5 (11.2–16.2)	<0.0001
Urbanization								
1	333	81763	40.7	112	339857	3.3	12.1 (9.78–15.0)	<0.0001
2	286	80295	35.6	78	330882	2.4	14.9 (11.6–19.1)	<0.0001
3	151	53418	28.3	43	218371	2.0	14.2 (10.1–19.9)	<0.0001
4+	162	49902	32.5	49	204794	2.4	13.4 (9.73–18.4)	<0.0001
Parental occupations								
White collar	585	168122	34.8	160	695044	2.3	14.9 (12.5–17.7)	<0.0001
Blue collar	145	45985	31.5	55	189081	2.9	10.7 (7.84–14.6)	<0.0001
Others	202	51270	39.4	67	209779	3.2	12.2 (9.24–16.1)	<0.0001

† Incidence rate, per 10000 person-years

Adjusted HR, adjusted hazard ratio controlling for sex, urbanization, and parental occupation.

Table 3 shows that the incidence rate of ROP increased with the decreased of birth weight in the twin cohort, from 7.09 per 10000 person-years in those with a normal birth weight to 1243.2 per 10000 person-years in those with birth weights < 1000 grams. There was a similar trend in the singleton cohort, from 0.47 per 10000 person-years to 1016.3 per 10000 person-years. Compared to singletons with normal birth weights, twins with normal birth weight had an adjusted HR of 14.9 (95% CI = 10.8–20.4) for ROP. The highest ROP risk was in twins with <1000 grams birth weight compare to singletons with normal birth weight (adjusted HR = 1475, 95% CI = 1053–2067).

**Table 3:** Incidence of retinopathy of prematurity by birth weight and Cox proportional hazards regression analysis estimated adjusted hazard ratio associated with of ROP using normal birthweight of singleton as reference.

	Singleton					Twin				
Birth weight, grams	N 111080	Event	Person-years	Rate†	Adjusted HR (95% CI)	N 27830	Event	Person-years	Rate†	Adjusted HR (95% CI)
Normal	109335	51	1080507	0.47	1.00	22156	153	215929	7.09	14.9 (10.8–20.4)
2000–2499	629	27	6732	40.1	93.5 (58.8–149)	2190	121	21846	55.4	121 (87.4–168)
1750–1999	346	44	3071	143.3	300 (201–450)	1438	161	12912	124.7	265 (193–363)
1500–1749	184	37	1406	263.1	513 (336–784)	915	134	7857	170.5	351 (254–485)
1250–1499	157	41	1095	374.3	689 (456–1040)	565	152	3914	388.3	708 (515–972)
1000–1249	116	37	650	569.2	892 (584–1636)	341	110	2107	522.1	820 (588–1144)
< 1000	113	45	443	1016.3	1202 (805–1796)	225	101	812	1243.2	1475 (1053–2067)

† Incidence rate, per 10000 person-years

Adjusted HR, adjusted hazard ratio controlling for sex, urbanization, and parental occupation.

Neonates with mechanical ventilation treatment had a higher ROP incidence in both cohorts, and were higher for twins than for singletons (Table 4). The highest incidence of 320.9 per 10000 person-years occurred to twins with invasive treatment, with an adjusted HR of 148 (95% CI = 118–185), compared to singletons without the invasive treatment.

**Table 4:** Incidence and Cox proportional hazards regression analysis estimated adjusted hazard ratio of retinopathy of prematurity associated with mechanical ventilation use at birth.

	Singleton						Twin					
	N	Event	Person-years	Rate†	Adjusted HR (95% CI)	P-value	N	Event	Person-years	Rate†	Adjusted HR (95% CI)	P-value
Mechanical ventilation												
None	110416	203	1087945	1.87	1.00		26691	717	256047	28.0	14.8(12.7–17.3)	<0.0001
Noninvasiv e ventilation	297	25	2973	84.1	47.9(31.6–72.6)	<0.0001	617	92	5498	167.3	88.6 (89.2–113)	<0.0001
Invasive ventilation	367	54	2986	180.8	88.9(65.8–120)	<0.0001	522	123	3833	320.9	148 (118–185)	<0.0001

† Incidence rate, per 10000 person-years

Adjusted for sex, urbanization, and parental occupation

Table 5 shows that ventilation treatments increased the risk of developing ROP. The impact appeared greater in twins than in singletons for infants with a birthweight of ≥ 1500 grams. On the other hand, singletons receiving invasive ventilation treatment had the highest ROP incidence for infants with a birthweight of <1500 grams.

**Table 5:** Incidence of retinopathy of prematurity by birth weight and Cox proportional hazards regression analysis estimated adjusted hazard ratio associated with mechanical ventilation.

	Singleton						Twin					
	N	Event	Person-years	Rate†	Adjusted HR (95% CI)	P-value	N	Event	Person-years	Rate†	Adjusted HR (95% CI)	P-value
Infants weight ≥ 1500 grams												
Noninvasive ventilation	253	15	2643	56.8	1.00		489	58	4606	125.9	2.14 (1.21–3.78)	0.009
Invasive ventilation	274	20	2499	80.0	1.29 (0.66–2.52)	0.46	270	54	2299	234.9	3.65 (2.06–6.47)	<0.0001
Infants weight < 1500 grams												
Noninvasive ventilation	44	10	330	303.2	4.68 (2.10–10.5)	0.0002	128	34	891	381.5	4.90 (2.66–9.02)	<0.0001
Invasive ventilation	93	34	487	697.5	7.58 (4.12–14.0)	<0.0001	252	69	1534	449.9	5.69 (3.25–9.95)	<0.0001

† Incidence rate, per 10000 person-years

Adjusted for sex, urbanization, and parental occupation

## Discussion

ROP is a commonly known complication for preterm birth infants, whom were most likely born at a low birth weight. Studies evaluating ROP risk for preterm babies associated with very low and/or extremely low birthweight may not make comparison with term babies or babies with normal birth weight which may lead to undermine the risk of ROP in clinical condition. Our study was to evaluate the ROP risk for low-birth-weight infants compared to normal birth weight singleton infants based on population data. Limited studies have compared the ROP risk between singletons and multiple birth neonates. [16– 19] We found even for infants with normal birth weight, twins had an adjusted HR of 14.9 to develop ROP compared with singletons. The adjusted HR increased to 1475 for extreme-low-birth-weight twins or to 1202 for extreme low birth singletons, compared to normal birth weight singletons, indicating the risk of developing ROP is extremely high for twin infants with extreme-low-birth-weight infants.

Previous studies may fail to find significant difference in the ROP risk between twins and singletons because of small sample sizes. With a large sample size, the present neonate population study showed that babies who developed ROP were much more common in twins than in singletons (3.35% vs. 0.25%) in Taiwan. This is mainly because the low-birth-weight infants were more prevalent in the twin cohort than in the singleton cohort (25.7% vs. 1.57%). The proportion of infants with extreme low birth weights of <1000 gm was also greater in twins than in singletons (0.81% vs. 0.10%). It is well known that preterm delivery rate and low birth weight rate are much greater in twins than in singletons. [22] The overall risk of developing ROP is thus much greater for twins than singletons. Our findings are in line with previous reports that birth weight and preterm births are important factors contributing to the development of the visual impairment.

An Israel study found that singletons are at a higher risk of advanced ROP than multiple gestations with birth weights of 1500 grams and less. [16] Our study also showed that the incidence rate of ROP in infants with birth weights of 1000–1999 grams was greater in singletons than in twins. However, for extreme-low-birth-weight infants, the incidence rate of ROP was greater in twins with the adjusted HR near 23% greater than singletons. This finding implies that twin births with an extreme low birth weight deserve greater attention for ROP prevention care.

The Swedish guidelines for ROP screening program evaluated 1744 preterm infants with a mean birth weight of 1239g (382–2615) and found 24.1% of them developed ROP.[23] A New York population-based retrospective cohort study comparing racial/ethnic groups among very preterm infants found that the Asian infants had an adjusted RR of 1.85 for ROP than white infants.[24] A Chinese study found 26.0% of very low birthweight infants developed ROP.[25] In this study, proportions of infants with a birth weight of <1000 grams developing ROP were even as high as 44.9% in twins compared to 39.8% in singletons.

It is well-known that excessive oxygen supply is an important risk factor involving in ROP pathogenesis and restricted oxygen delivery may reduce the ROP development.[26– 28] Recent meta-analyses reported that extreme low birth weight or extreme preterm birth infants with exposure to a low oxygen saturation (85–89%) had near 25% reduced risk compared to exposure to a high oxygen saturation (91–95%).[26, 27] In the present study, the ROP incidence rate was also lower for infants exposed to non-invasive ventilation use at births than those exposed to invasive ventilation use. However, the incidence remained greater in twins than in singletons in both types of ventilation, particularly for those with a birth weight of less than 1500 gm. The non-invasive ventilation use had a greater contribution in reducing the ROP incidence for singletons (56.5%) than for twins (15.2%) among those with a birth weight of less than 1500 gm. It is not clear why the invasive ventilation use has relatively a greater association for singletons than for twins among very low-birth-weight infants. Future research may explore risk of ROP associated with the invasive ventilation use based on severity of ROP between singletons and multiple births. Finally, twin–twin transfusion syndrome as a possible risk factor for the development of retinopathy of prematurity has also been noted. Infants with twin–twin transfusion syndrome are at a higher risk of developing ROP than those without the syndrome, even they are in full term [29]. All these risk factors described are recommended for a special awareness in ROP screening for infants.

### Strength and limitation

Our study has strengths of reducing selection bias on its nationwide population-based retrospective cohort design to exam the ROP risk between the twins and the singletons. Moreover, we established study cohorts with large sample sizes, matched by sex, birth year, urbanization level of residential area and parental occupation, to eliminate confounding effect.

However, this study has limitations. First, information on most severity stage of ROP and the gestational age at birth was unavailable in the claims data. In a review, Fuchs and Senat reported that preterm birth account for almost 50% of twin pregnancies [30]. Twins are more likely born premature with a shorter gestational age and lower birth weight, with a higher portion under the invasive mechanical ventilation treatment. They are thus more likely to have ROP at more severe stage. Twin infants might receive greater pediatric attention at birth and after birth as they were likely admitted to the neonatal intensive care unit. Second, in the clinical practices in Taiwan, non-invasive ventilation and invasive ventilation are not exactly similar to low oxygen saturation treatment (85–89%) and high oxygen saturation treatment (91–95%). The former means the way of oxygen therapy; the latter, target for this therapy. The oxygen saturation of the newborns with non-invasive ventilation are used to achieving 90%; otherwise, a more progressive approach are suggested, such as invasive ventilation. However, this does not mean the utilization of ventilation conversely to oxygen saturation treatment. Finally, we randomly selected one infant from the twins without differentiating identical twins and non-identical twins in this study. We, therefore, could not compare the ROP risk between twin siblings and the risk associated with twin–twin transfusion syndrome.

In summary, the overall incidence of ROP is higher in twin birth infants because of higher portion of low-birth-weight infants with, which could be mainly due to preterm birth. Twins are at a higher risk of ROP after being cared with mechanical ventilation. Oxygen management should be approached with caution for low-birth-weight infants of both singletons and twins. Additional work is needed to identify the adequate strategies for ROP prevention.
